# MiR-223-5p works as an oncomiR in vulvar carcinoma by *TP63* suppression

**DOI:** 10.18632/oncotarget.10247

**Published:** 2016-06-23

**Authors:** Beatriz de Melo Maia, Iara Santana Rodrigues, Erica Mie Akagi, Nayra Soares do Amaral, Hui Ling, Paloma Monroig, Fernando Augusto Soares, George Adrian Calin, Rafael Malagoli Rocha

**Affiliations:** ^1^ Molecular Morphology Laboratory, Anatomic Pathology Department, AC Camargo Cancer Center, São Paulo, Brazil; ^2^ Department of Experimental Therapeutics, The University of Texas, MD Anderson Cancer Center, Houston, TX, USA; ^3^ The Center for RNA Interference and Non-Coding RNAs, The University of Texas, MD Anderson Cancer Center, Houston, TX, USA; ^4^ Gynecology Laboratory, Gynecologic Department Federal University of São Paulo, São Paulo, Brazil

**Keywords:** vulvar cancer, microRNAs, cellular assays, hsa-miR-223-5p, TP63

## Abstract

MiR-223-5p has been previously mentioned to be associated with tumor metastasis in HPV negative vulvar carcinomas, such as in several other tumor types. In the present study, we hypothesized that this microRNA would be important in vulvar cancer carcinogenesis and progression. To investigate this, we artificially mimicked miR-223-5p expression in a cell line derived from lymph node metastasis of vulvar carcinoma (SW962) and performed *in vitro* assays. As results, lower cell proliferation (*p* < 0.01) and migration (*p* < 0.001) were observed when miR-223-5p was overexpressed. In contrast, increased invasive potential of these cells was verified (*p* < 0.004). *In silico* search indicated that miR-223-5p targets *TP63*, member of the TP53 family of proteins, largely described with importance in vulvar cancer. We experimentally demonstrated that this microRNA is capable to decrease levels of p63 at both mRNA and protein levels (*p* < 0.001, and *p* < 0.0001; respectively). Also, a significant inverse correlation was observed between miR-223-5p and p63 expressions in tumors from patients (*p* = 0.0365). Furthermore, low p63 protein expression was correlated with deeper tumor invasion (*p* = 0.0491) and lower patient overall survival (*p* = 0.0494). Our study points out miR-223-5p overexpression as a putative pathological mechanism of tumor invasion and a promising therapeutic target and highlights the importance of both miR-223-5p and p63 as prognostic factors in vulvar cancer. Also, it is plausible that the evaluation of p63 expression in vulvar cancer at the biopsy level may bring important contribution on prognostic establishment and in elaborating better surgical approaches for vulvar cancer patients.

## INTRODUCTION

Vulvar carcinoma, despite its rarity, ranks as the fourth most common gynecologic malignancy in women and was responsible for 4,850 new cases and 1,030 deaths last year in the United States [1, 2]. Treatment is mainly surgical and despite a less radical surgery can be employed in a subset of localized lesions, psychosexual morbidity is still an important matter [3, 4]. Although great efforts are being made to a better understanding of this disease at the biological level, new prognostic and predictive factors continue highly required in vulvar cancer.

Numerous epidemiological, clinical, pathological and molecular studies indicate the existence of two etiologies of vulvar carcinomas: in the first group, women are older–from 55 to 85 years old–and develop lichen sclerosus associated carcinomas, squamous hyperplasia, differentiated vulvar intraepithelial neoplasia (VIN), and TP53 mutations, not being these tumors directly associated with HPV infection [5–7]. The second group consists of younger women, aged between 35 and 65 years old, in which tumors are often related to infection with high-risk HPVs, such as HPV 16, 18 and 33 [5, 7].

MicroRNAs (small non-coding RNAs of approximately 22nt length) participate as controllers of several human disease processes including virtually all types of cancer and pose as clinical promises on the biomedical field [8, 9]. They inhibit protein translation in a very complex manner by binding to the 3′UTR portion of their target mRNAs, acting at a posttranscriptional level [8]. The literature also reports that microRNAs can also target 5′UTR region [10], gene promoter regions [11], proteins [12], DNA [13] and Toll-like receptors, TLR [14]. MicroRNA-microRNA binding [15], microRNA-pseudogenes interaction [16], as well as direct repression of the epigenetic machinery – such as DNA methyltransferases DNMT3A and DNMT3B [17] –have also been well demonstrated. All these illustrate the complexity surrounding microRNA functions, but the employment of microRNAs with a clinical purpose is widely expected in many fields of medicine.

Recently, our group demonstrated that altered expression of miR-223-5p was associated to the presence of nodal metastasis in patients with vulvar cancer [18]. In fact, this microRNA has emerged as a putative predictor of tumor invasiveness and metastasis, [19–21] as well as recurrence [22, 23] in many tumor types. Herein, we further explored the role of miR-223-5p in a metastatic vulvar cancer cell line (SW962) and evaluated one of its targets in a cohort of vulvar cancer patients in order to check if this microRNA would have a potential impact on vulvar cancer behavior *in vitro*.

In the present study, we found that artificial expression of miR-223-5p through mimetic oligonucleotides transfection decreased cell proliferation and migration but, at the same time, increased the invasive potential of the cells. We also provide evidence that this microRNA targets TP63 and is capable to decrease this target at both mRNA and protein levels. We further demonstrated that low p63 protein expression in samples from patients was correlated with deeper tumor invasion and lower overall survival and that p63 loss may stimulate tumor progression. Altogether, these results indicate that miR-223-5p can modulate a putative pathological mechanism of tumor invasion and can be a promising prognostic factor and therapeutic target in vulvar cancer.

## RESULTS

### MiR-223-5p is low expressed in SW962 cells

**Figure 1:**
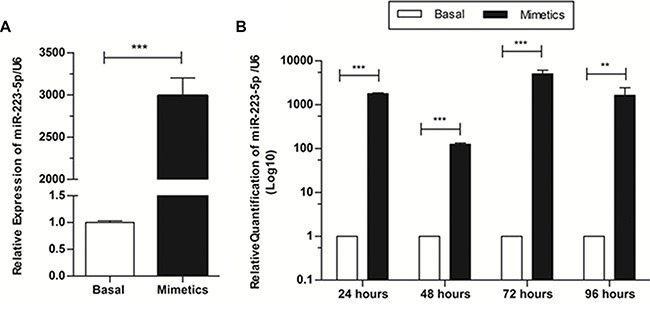
Expression levels of miR-223-5p are increased when mimetic oligonucleotides are transfected onto SW962 cells and this increment lasts at least 96 hours after transfection (**A**) Relative expression of miR-223-5p was significantly increased when mimetics were added, compared to non-transfected – basal cells, detected by qRT-PCR. (**B**) MiR-223-5p expression remains significantly higher on transfected cells compared to basal SW962 for at least 96 hours post transfection. ** on behalf of *p* ≤ 0.01, and ***p ≤ 0.001.

Basal expression levels of miR-223-5p in SW962 cell line were found to be very low by qRT-PCR (CT values ranging from 37.82 to 39.98). So, we proposed the evaluation of the functional role of this microRNA through the use of mimetics. In order to investigate if artificial increment of miR-223-5p expression would have a biological function on SW962 cells, we performed *in vitro* assays in conditioned culture medium with or without addition of miR-223-5p oligonucleotides. The expression of miR-223-5p was significantly increased when mimetics were added, compared to basal level (*p* = 0.0002) (Figure 1A). We also checked the duration of this increase and if it would last enough to have several assays performed (approximately 96 hours) and we observed that the microRNA expression remained significantly higher on transfected cells compared to basal SW962 during the time-points (*p* < 0.0001), with a peak of expression at 72 and 96 hours (Figure 1B).

### Artificial expression of miR-223-5p reduced proliferation on SW962 cells

**Figure 2:**
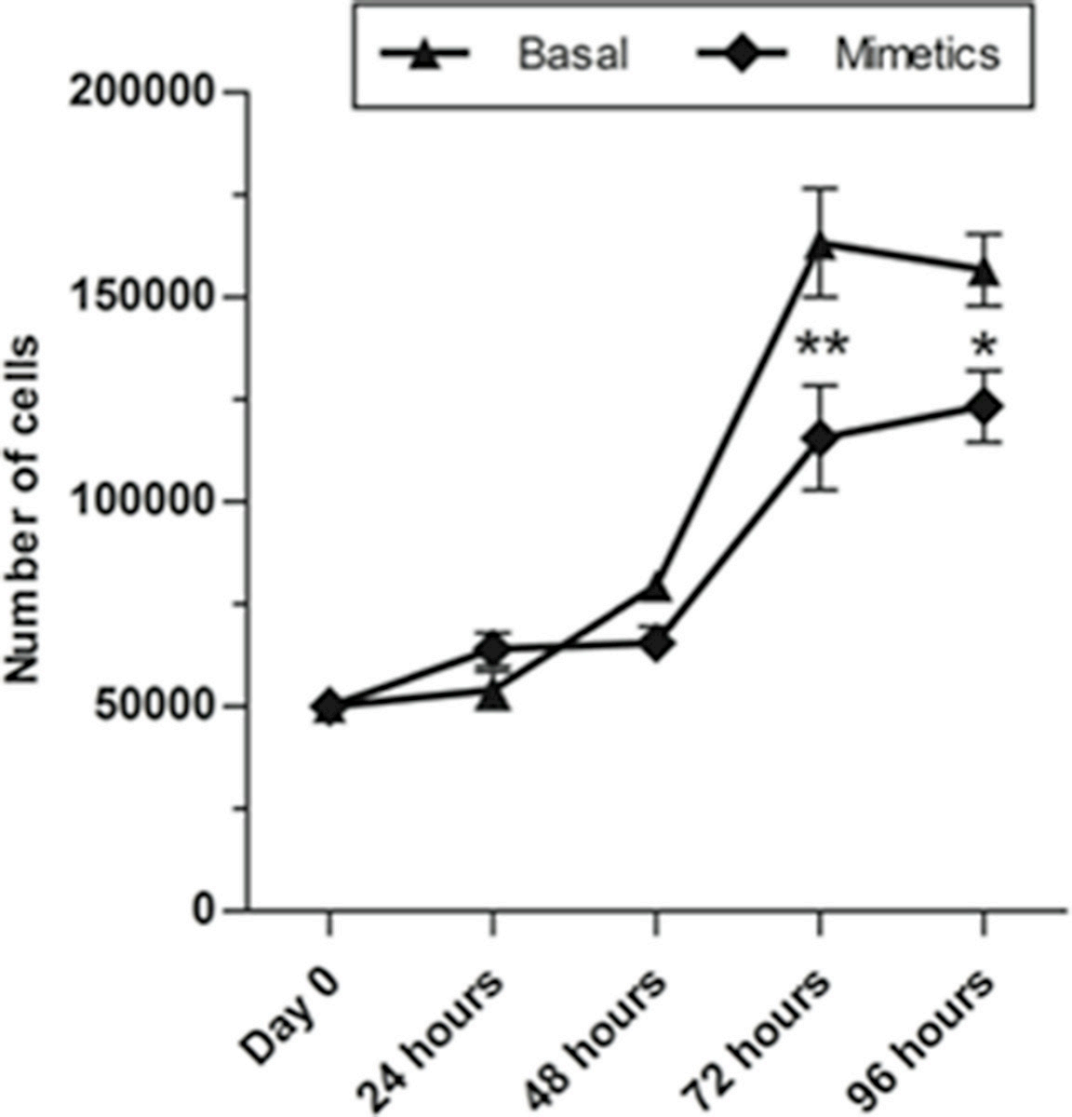
MiR-223-5p mimetics transfection inhibited proliferation of SW962 cells Proliferation rates of SW962 cells were significantly reduced after mimetics microRNA were transfected compared to basal non-transfected cells at 72 hours and 96 hours post-transfection. ** on behalf of *p* ≤ 0.01 and * on behalf of *p* ≤ 0.05.

Cell proliferation assay was employed to determine if miR-223-5p would be able to alter this key factor of malignant progression and was evaluated by automated cell number quantification. MiR-223-5p significantly decreased the number of proliferation cells 72 and 96 hours post-transfection in comparison with the control groups (basal cells, non-transfected), indicating the inhibition of SW962 cell proliferation (*p* ≤ 0.01 and *p* ≤ 0.05, respectively) (Figure 2).

### MiR-223-5p increased invasive but not migratory potentials of SW962 cells

**Figure 3:**
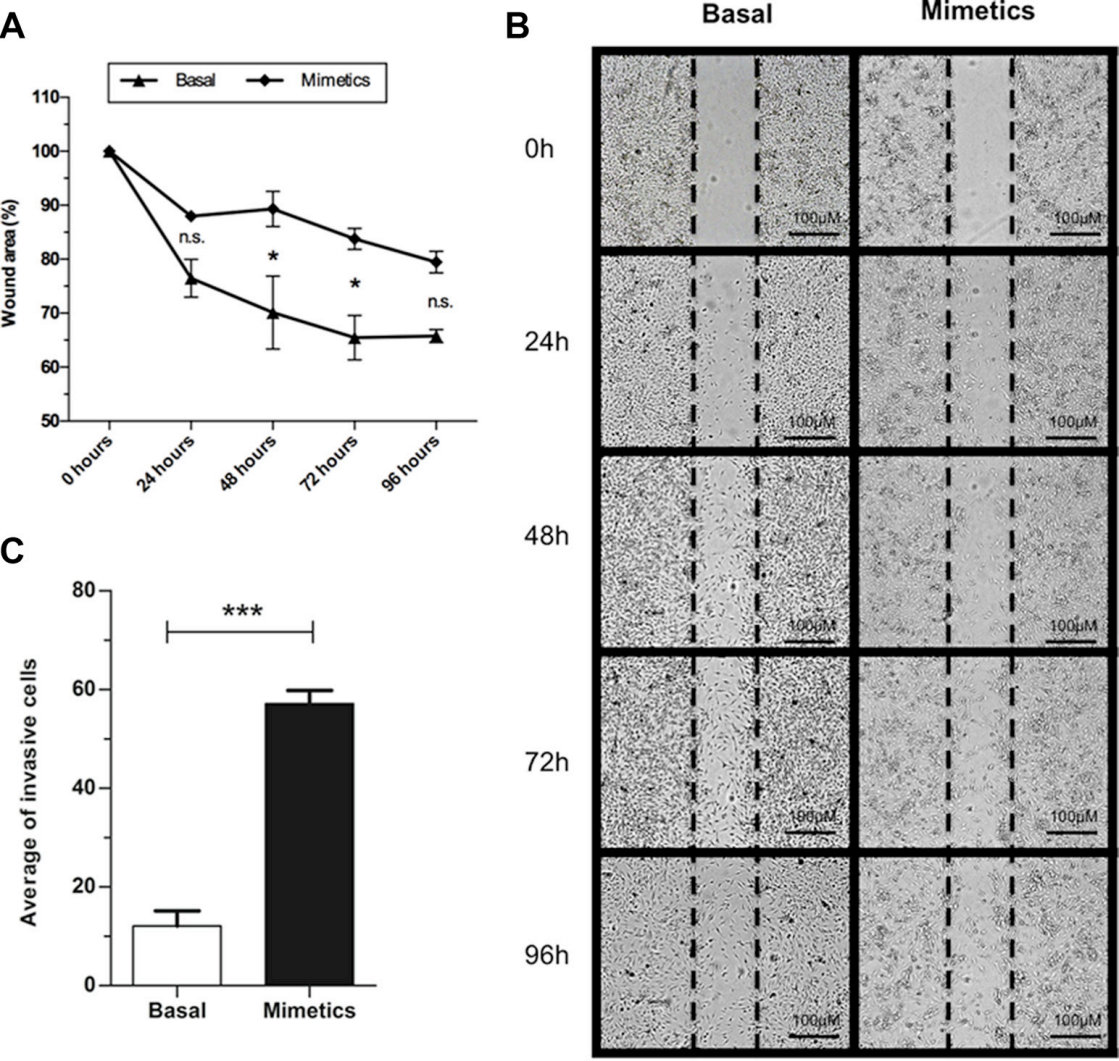
Invasion and wound-healing assays for basal and mimetic-transfected SW962 cells (**A**) Cells transfected with mimetic microRNA presented lower migration (higher wound area) compared to basal SW962 cells at 48 and 72 hours post-transfection. (**B**) Illustrative image of wound-healing assay at five time-points. (**C**) Higher number of invasive cells was observed when cells were transfected with miR-223-5p. *** on behalf of *p* ≤ 0.001 and * on behalf of *p* ≤ 0.05, n.s. on behalf of non-significant values.

Wound-healing assay showed that miR-223-5p led to a lower wound-healing cell migration after 48 and 72 hours in comparison to basal cells control group (*P* < 0.001) (Figure 3A and 3B). In contrast, cells transfected with miR-223-5p for 24 h exhibited an increased effect on cell invasion though the Matrigel chamber in comparison with control ones (*P* = 0.0004) (Figure 3C).

### *TP63* as a direct target of miR-223-5p

**Figure 4:**
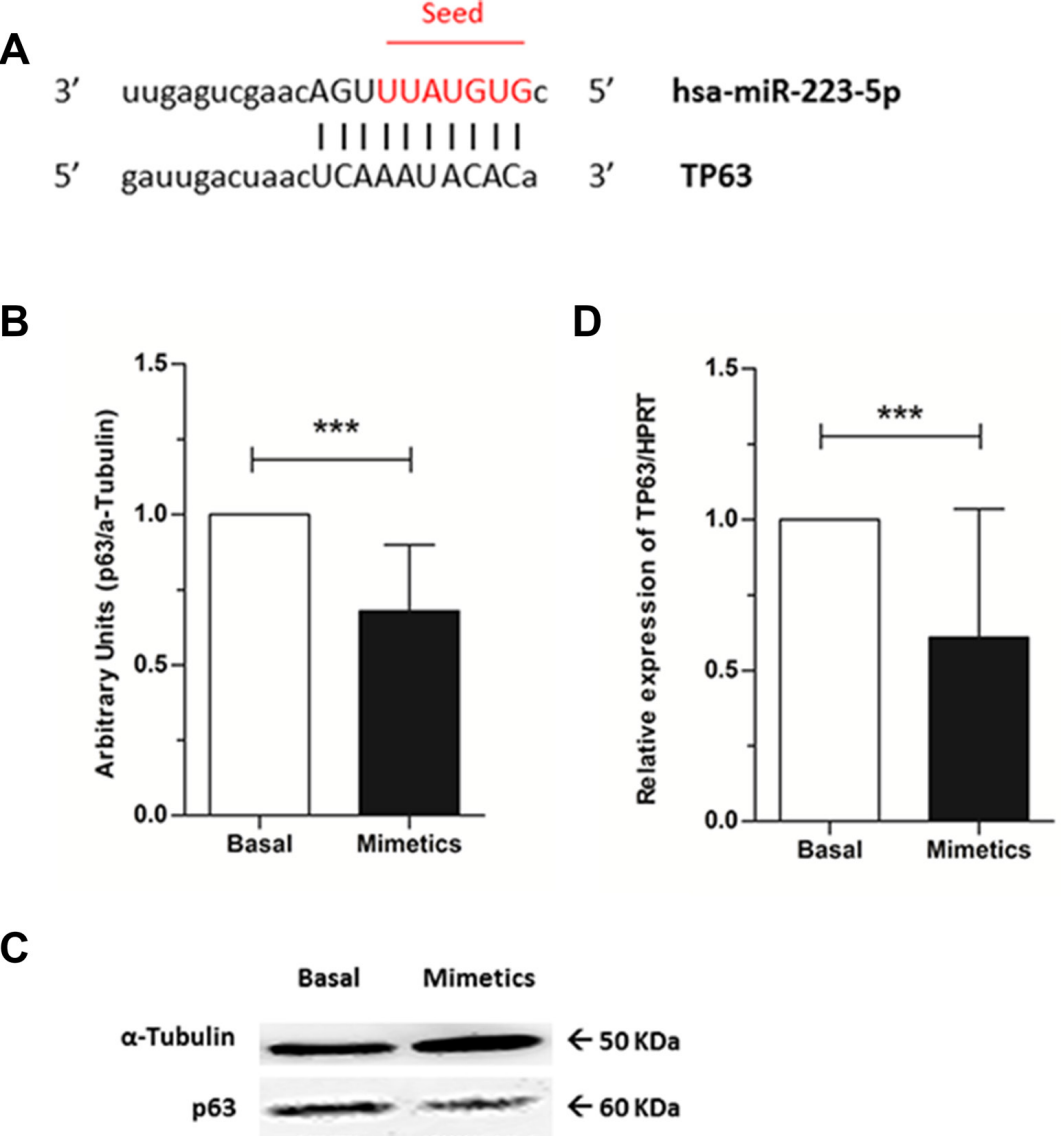
TP63 as a target for hsa-miR-223-5p and p63 protein levels evaluation (**A**) TP63 mRNA (bottom) contains a binding site from positions 956-977 for hsa-miR-233-5p (top). Mature microRNA is aligned with TP63 mRNA and hsa-miR-223-5p seed sequence is highlighted in red. (**B**) A significant reduction of p63 protein levels was observed when mimetics were added to SW962 cell line. Quantitative analysis and (**C**) illustrative image, being α-Tubulin was used as internal control. (**D**) Relative expression of TP63 by qRT-PCR normalized to HPRT was significantly lower on mimetics transfected cells compared to basal SW962 cells. *** on behalf of *p* ≤ 0.0001.

We then sought to identify a target not previously validated for miR-223-5p and whether the increment of microRNA expression would be able to repress this target. Using target gene prediction algorithms, we obtained *TP63* as a putative target for hsa-miR-223-5p. Our search identified that the 3′UTR *TP63* mRNA contains a complimentary binding site for hsa-miR-223-5p seed region (Figure 4A).

By western blot, we observed that p63 protein levels were significantly decreased when mimetics were added to the cells (*p* < 0.0001) (Figure 4B and 4C) and by qRT-PCR, lower expression of *TP63* mRNA was obtained on microRNA overexpressing cells when compared to basal ones (*p* < 0.0001) (Figure 4D).

### P63 is located in the nuclei of vulvar carcinoma tissues and SW962 cells

**Figure 5:**
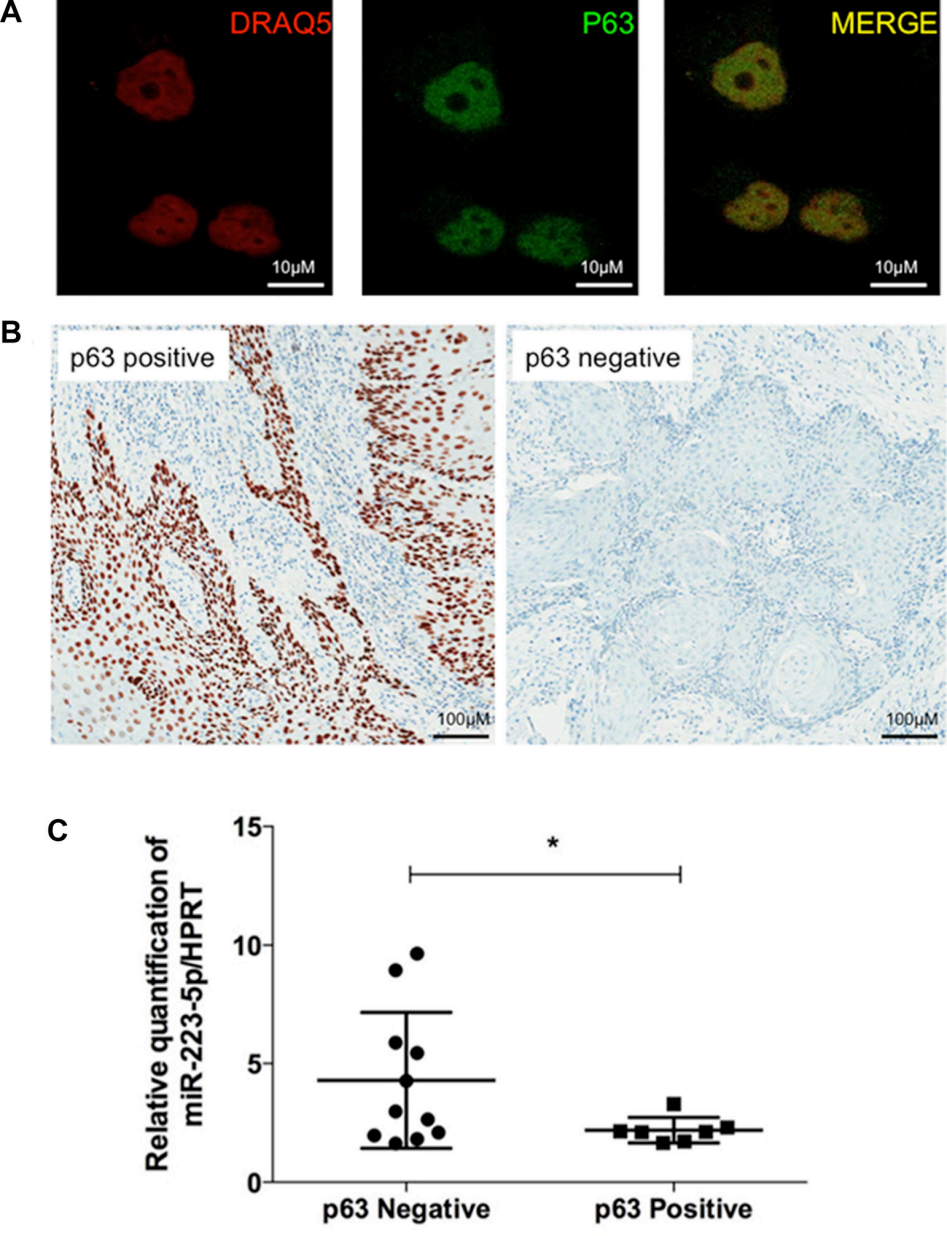
Protein p63 is located in the nuclei of vulvar carcinoma tissues and SW962 cells and is inversely correlated to miR-223-5p expression (**A**) P63 is located in the nuclei of SW962 cells by immunofluorescence assay. From left to right, nuclei of vulvar SW962 cells are stained with DRAQ5 (Red); anti-p63 antibody is labeling cells for p63 protein (Green); merged image demonstrate nuclear expression of p63 (yellow). Magnification: 400×. (**B**) Representative images of p63 immunostaining (brown) in FFPE vulvar tumors, positive case (HScore < 150) on the left side and negative case (HScore ≥ 150) on the right side. Magnification: 200×. (**C**) Scatter dot plot demonstrate the distribution of patients with p63 positive and negative tumors versus miR-223-5p expression levels. Patients with negative p63 (left) presented statistically significant higher expression of miR-223-5p compared to the patients with p63 positivity * on behalf of *p* < 0.05.

Immunofluorescence assay demonstrated that p63 is solely located in the nuclei of both basal and transfected SW962 cells (Figure 5A). Similarly, all immunohistochemically stained cases demonstrated purely nuclear p63 localization (Figure 5B). Positive expression of p63 (HScore ≥ 150) in tumor samples was observed in 38.89% of cases versus 61.11% negativity.

### miR-223-5p is overexpressed in low p63 samples

We were able to demonstrate by qRT-PCR that there is an inverse correlation between p63 protein expression (by IHC) and miR-223-5p expression (by qRT-PCR). Patients with negative p63 presented significantly higher expression of miR-223-5p compared to patients with high p63 expression by IHC (*p* = 0.0365, Figure 5C).

### Decreased p63 expression is associated with lower survival and deeper tumor invasion in vulvar cancer patients

**Figure 6:**
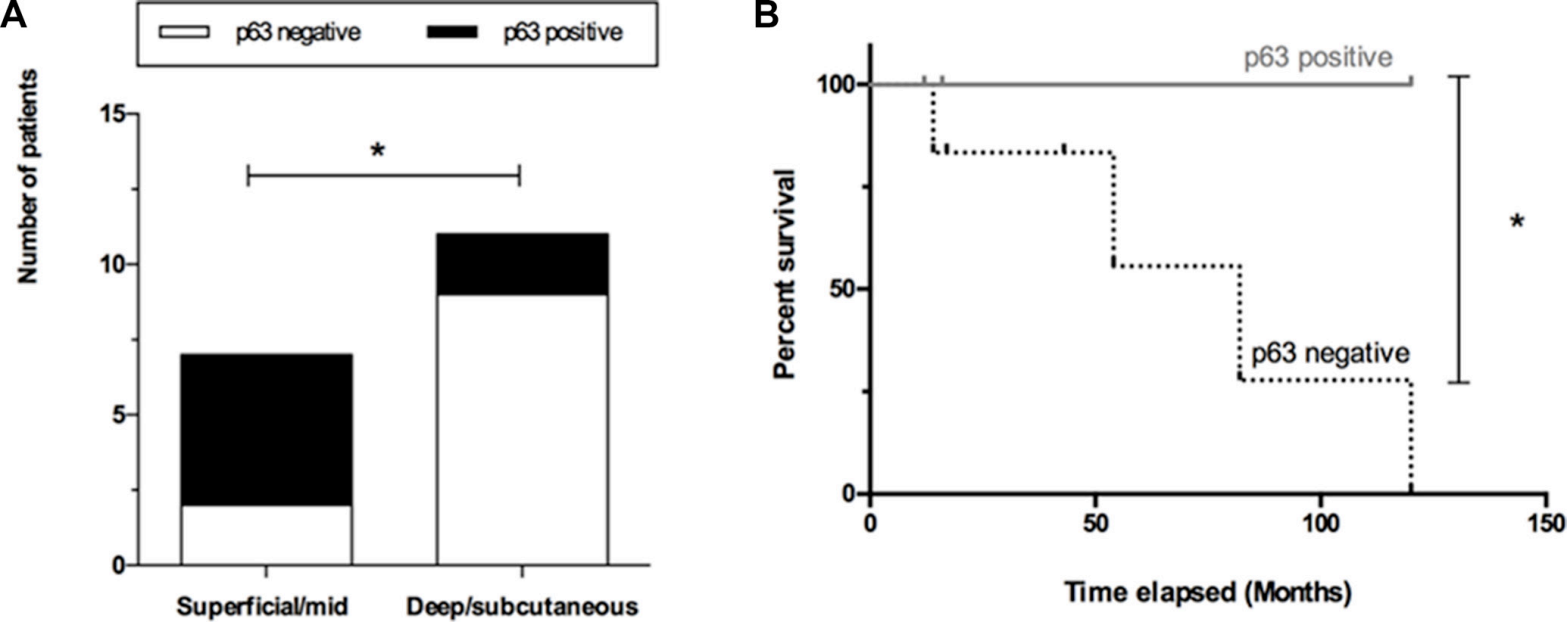
P63 expression correlates with patient outcome (**A**) Lack of p63 expression (white bars) was correlated with deeper tumor invasion (deep dermis or subcutaneous infiltration), (**B**) and lower survival rates (dashed line). * on behalf of *p* = 0.05.

Low expression of p63 in patients with VSCC correlates with patient outcome. We found an association between low p63 expression (HScore < 150) with deeper tumor invasion (deep dermis or subcutaneous infiltration) (*p* = 0.0491, Figure 6A) and lower survival rates (*p* = 0.0494, Figure 6B). Other features such as tumor grade, HPV infection, nodal metastasis and perineural/vascular invasions did not present any statistically relevant associations with p63 expression by IHC (Supplementary Table S1).

### P63 negativity is associated with tumor progression

**Figure 7:**
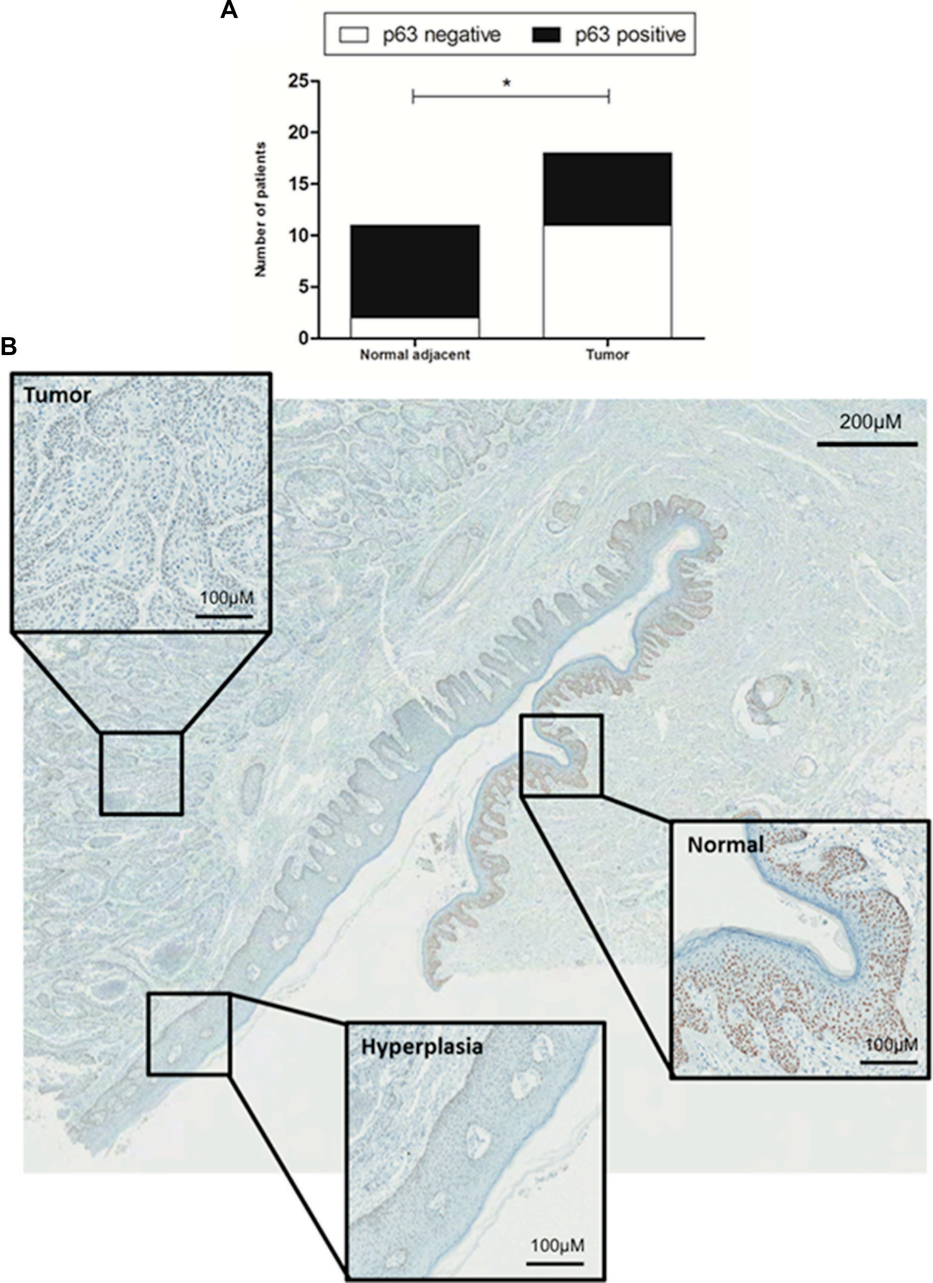
Immunohistochemical evaluation of normal tissue *versus* hyperplasia and tumor (**A**) Normal adjacent tissue (*n* = 11) demonstrated a significant overexpression of p63 protein (black bars) compared to tumor (*n* = 18). (**B**) Progression of normal epithelium to hyperplastic epithelium to tumor demonstrates loss of p63 expression. Whole figure magnification: 20×; Boxes magnification: 200× * on behalf of *p* = 0.05.

Another interesting immunohistochemical finding was obtained in relation to normal tissue adjacent to the tumor evaluation. IHC scoring of normal epithelia was achievable in eleven (out of 18) cases evaluated in the present work, and 81.8% of the normal tissues presented p63 immunostaining (versus 18.2% of negativity). When compared to tumor samples, normal adjacent tissue demonstrated significant overexpression of p63 protein (*p* = 0.0241, Figure 7A). Furthermore, the progress of normal epithelium to hyperplastic process tumor was associated with loss of p63 expression (Figure 7B).

## DISCUSSION

The first mention of microRNA differential expression in vulvar cancer was provided by our group on a previous study [18] and miR-223-5p appeared as an important microRNA associated with metastasis in HPV negative vulvar samples. In fact, miR-223 arose as a recognized tumor invasiveness and metastasis predictor in several tumor types: its expression was correlated with metastasis in breast [19], gastric [20], colorectal [24] and hepatocellular [25] carcinomas, and osteosarcoma [26]. Likewise, miR-223 was correlated with recurrence in ovarian [22] and hepatocellular [23] carcinomas. Because of this, miR-223 is considered at the present time as an oncomiR, i.e. member of tumor promoting microRNAs [21].

In the present study, we hypothesized that microRNA miR-223-5p would have a potential role on vulvar cancer behavior. To achieve this, we artificially mimicked microRNA expression in an HPV negative vulvar cancer cell line – SW962, derived from a lymph node metastasis of vulvar carcinoma – and performed *in vitro* studies. Our findings confirmed other recent reports that have documented the possible role of this microRNA in tumor invasiveness by demonstrating that miR223-5p expression increased the invasive ability of SW962 cells, which suggests that a higher expression of miR-223-5p was capable to amplify their metastatic potential. After *in silico* study, we identified p63 as potential mRNA target for miR-223-5p, evaluated p63 expression at both protein and mRNA levels *in vitro* and, finally tested if this target presented any association with patient outcome using IHQ on FFPE samples.

We provide experimental evidence that overexpression of miR-223-5p inhibited the proliferation of SW962 cells. This suggests that miR-223-5p exhibits an anti-proliferative activity in the vulva, and this is in line with other studies in the literature in other cancer cell lines. Overexpression of miR-223 was capable to block myeloid cell proliferation through E2F1 inhibition [27] and miR-223 was described as a suppressor of cell proliferation in malignant pleural mesothelioma cells through *STMN1* targeting [28]. Inhibition of cell proliferation through *IGF-1R* targeting was also demonstrated [29, 30].

After, our results also demonstrate that overexpression of miR-223-5p led to a more invasive phenotype to SW962 cells. Similarly, Li et al. (2011) [24] shown that miR-223 was able to stimulate non-metastatic gastric cancer cells to invade by targeting a tumor suppressor gene named *EPB41L3*. Wei et al. (2014) [31] also found accelerated invasive ability of Prostate Cancer cell lines when miR-223-3p were artificially added to the cells, but in this case through *SEPT6* targeting.

Current knowledge about vulvar cancer etiology indicates two distinct pathways for this disease, one associated with HPV infection and the other associated with TP53 mutation [5–7]. Since all commercially available vulvar cancer cell lines are HPV negative and since the previously demonstrated association between miR-223-5p expression and nodal metastasis was obtained on HPV negative samples, we hypothesized that it would be ideal to check if members of TP53 family were targeted by miR-223-5p. *TP63*, member of *TP53* family of tumor suppressor genes and largely described with importance in vulvar cancer, was identified as a target for miR-223-5p in three prediction algorithms and thus, further explored as a target for miR-223-5p *in vitro* and in relation to patient's clinicopathological features. P63 protein is essential for maintaining keratinocytes proliferation; and loss of p63 leads to cell cycle arrest and lower proliferation rates [32, 33]. Since miR-223-5p decreases p63 expression, we believe that loss of p63 may explain the lower proliferation rates of transfected cells compared to basal SW962 cells.

The complimentary mRNA-microRNA linkage within mRNA 3′UTR recruits RNA-induced silencing complex (RISC) silencing complex, protein expression is decreased and mRNA is degraded [34, 35]. In our work, increased invasive ability of SW962 cells may have been given to the post-transcriptional suppression of p63 by miR-223-5p. Therefore, the artificial expression of miR-223-5p in the cells may have led to p63 decreased expression– at both protein and mRNA levels, as shown by western blot and qRT-PCR, respectively. Several authors found that p63 knockout, most predominantly the ΔNp63 isoform, was directly linked to an increase of invasive profiles in cancer [36–39]. Koga et al. (2003) [40] found that more aggressive (high-grade/invasive) urothelial carcinomas presented a frequent decrease or loss of ΔNp63. Suppression of p63 resulted in stimulation of genes associated with invasion and metastasis [41] and epithelial-to-mesenchymal transitions (EMT) [42]. Taking all this into account, we believe that increased invasion of SW962 transfected cells took place in the absence of p63.

We found a total contradictory result in regard to migration and miR-223-5p. While increased invasion of SW962 cells were observed in the presence of the mimetic oligonucleotide, the migratory ability of the cells was decreased. However, it is important to keep in mind that, although highly important for the metastatic processes, migration and invasion do not always walk together. In fact, in terms of cell biology, migration and invasion are two separate entities [43, 44]. While migration is defined as the movement of cells from one place to another on a two-dimensional environment, invasion is characterized as the movement of the cells through three-dimensional matrix. In this situation, cells are required to not only modify their shape and adhesions, but to proteolytically cleave adjacent extracellular matrix structures and for that, they use proteases which can be intracellular matrix metalloproteinases (MMP), surface-localized or secreted [44–46]. In fact, cells that underwent EMT were shown to migrate less than pre-EMT cells, indicating that migration and invasion processes can be disjoined [44]. Kolli-Bouhafs and colleagues (2014) demonstrated that mutation on FAK kinase (Tyr397) induced decreased migration but increased invasive properties in B16F10 melanoma cells [47]. All these lead us to infer that increased invasion does not have to be accompanied by increased migration.

TP63 was identified as a putative target for miR-223-5p in three prediction algorithms. This gene leads to a protein which is a member of the p53 family and that, similarly to all members of this family, presents three functional domains: an N-terminal transactivation–TA domain, a central DNA-binding domain and an oligomerization domain [39, 48, 49]. TP63 gene possesses two promoters generating two groups of p63 isoforms, one containing a functional N-terminal transcription-transactivating domain (TA) and the other lacking the TA domain, presenting an N-terminally truncated form (ΔN). Both types can go through alternative splicing at the C termini into three different variants (α, β or γ) [39, 48–51]. P63 is implicated in cell development [33, 52] and particularly ΔNp63α isoform is highly expressed in basal layers of stratified epithelium, being shown as essential for maintaining the integrity of these tissues [48]. Interestingly, ΔNp63α was demonstrated to participate in the recruitment of RNA Polymerase II to the promoter of the miR-205 host gene (miR-205HG), coordinating the transcription of both miR-205HG and miR-205 [49]. MiR-205 is repressed by both TAp63 and ΔNp63 and, in the presence of prostate cancer metastases, loss of both p63 and miR-205 were observed [53]. Also, TAp63 isoform is capable to inhibit the expression of Dicer, responsible for pre-microRNA cleavage, demonstrating great importance in metastasis suppression through global microRNA expression [49, 54].

Our data demonstrated that the expressions of p63–at both protein and mRNA levels–were significantly reduced by miR-223-5p in SW962 cells. Altogether, our results demonstrate that miR-223-5p is capable to effectively bind to TP63 mRNA and knock down its expression, probably through degradation of its mRNA (based on their totally complimentary binding fashion) and to the best of our knowledge, this is the first study to experimentally describe TP63 as a target for miR-223-5p.

In contrast to various studies evaluating tumor migration and p63 [25, 39, 41], our results indicate lower migratory rates of the cells transfected with miR-223-5p mimetic oligonucleotides. According to Li et al. 2011 [20], miR-223 was capable to stimulate the migration of several non-metastatic gastric cancer cell lines. Importantly, SW962 is a cell line derived from a lymph node metastasis of vulvar cancer. Consistently in line with this, Schaeffer et al. (2014) [44] state that improved migration is not a phenotypic prerequisite of EMT.

Next, we sought to evaluate if there was an association between miR-223-5p and p63 levels in patient tumors, and if p63 protein expression was correlated with patient outcomes. As expected, we found an inverse correlation between p63 loss and higher miR-223-5p expression. This indicates that p63 is also a target for this oncomiR in cancer patients. We found p63 expression restricted to the nuclei of the cells both by immunohistochemistry (for FFPE samples) and immunofluorescence (for SW962 cell line), which corroborates with its nuclear transcriptional activity and with others in the literature, inclusively in vulvar cancer [55, 56]. Importantly, the antibodies used for both techniques and western blot detect all six isoforms of p63.

Low p63 expression was significantly correlated with higher tumor invasion. In pathology, invasion is characterized as the infiltration of malignant tumor cells into the underlying interstitial tissues [43] and for statistical purposes we divided tumor invasion as compromising superficial/mid dermis or deep dermis/subcutaneous. Molecularly speaking, p63 activates cell-cell adhesiveness through Perp, a tetraspan membrane protein critical for desmosomal adhesion [57, 58]. Decreased p63 expression was demonstrated in the progression of superficial to invasive bladder tumors, but certain invasive tumors sustained widespread p63 expression [59]. Lodillinsky and collaborators (2015) [60] showed that the transition from ductal carcinoma *in situ* (DCIS) to invasive breast carcinoma requires membrane-type 1 (MT1) matrix metalloproteinase (MMP)/p63 axis activation, reinforcing p63 as an inhibitor of tumor invasiveness.

We noted that p63 was largely expressed on normal adjacent epithelia, but this expression was continuously lost as cells progressed to hyperplasia and tumor. In fact, p63 is expressed on the basal cell component of epithelia from a variety of tissues [50] and seems to repress differentiation [61] and tumor progression [38, 40, 59, 62].

Our results also demonstrated that patients with low p63 tumors presented worst survival rates. Similar results have previously been described on the literature [58, 63–65]. Altogether, our results allow us to indicate the loss of p63 as a possible marker of worst prognosis in vulvar cancer.

It is noteworthy that HPV, a major risk factor for vulvar cancer [4, 66], requires p63 for its late viral functions [67]. While SW962 cells are HPV negative [68], almost 40% of our FFPE samples were demonstrated to be HPV positive. However, no association between p63 expression and HPV status was achieved. Lack of p63 expression and HPV correlation has been made known before [67]. All this may be explained because, contrary to p53, p63 is not inhibited by viral HPV oncoproteins such as E6 [69].

Importantly, our results points out miR-223-5p overexpression as a putative pathological mechanism of tumor invasion behaving as an oncomiR. Taking this into account, miR-223-5p poses as having a promising therapeutic potential. The inhibition of this oncomiR in high miR-223-5p/low p63 patient tumors through artificial microRNA silencing mechanisms, such as antimiR nucleotides or microRNA sponges [70] would be a tool for repressing tumor invasiveness, and may represent an opportunity for vulvar cancer treatment. Future studies needs to be performed in this direction aiming not only to validate our findings in a bigger cohort but also create *in vitro* and *in vivo* inhibition models for miR-223-5p in vulvar cancer.

The reader is cautioned that the findings reported on this study regarding p63 expression are based on a randomly selected small cohort, and the data points out for future directions on vulvar cancer prognosis. Also, the lack of a normal vulvar cell line makes difficult the comparison of miR-223-5p basal levels between normal vs. tumor *in vitro*.

In sum, our results show for the first time *TP63* as a target for miR-223-5p and suggest that miR-223-5p is associated to vulvar cancer oncogenesis at least in parts through p63 suppression. Taken together, our findings indicate that miR-223-5p is capable to inhibit proliferation and migration, *in vitro*, at least in parts by suppressing p63 expression and/or degrading *TP63* mRNA. We also point out miR-223-5p overexpression as a putative pathological mechanism of tumor invasion through p63 repression, acting as promising therapeutic target. Also, it is plausible that the evaluation of p63 expression in vulvar cancer at the biopsy level may bring important contribution on prognostic establishment and in elaborating better surgical approaches for vulvar cancer patients. Our study highlights the complexity surrounding p63 biological mechanisms and the importance of this protein as a prognostic factor in vulvar cancer.

## MATERIALS AND METHODS

### Cell line and culturing conditions

SW962 vulvar cancer cell line (derived from a vulvar carcinoma lymph node metastasis, HTB-118) was obtained from the American Type Culture Collection - ATCC (Manassas, VA, USA) and maintained routinely with RPMI 1640 media, (GIBCO^®^, Life Tecnologies, Carlsbad, CA, USA) supplemented with 10% fetal bovine serum (FBS; GIBCO^®^, Life technologies, Carlsbad, CA, USA) and 100 mg/mL Garamycin. Cells were cultivated at 37°C with 5% CO_2_ humidified atmosphere.

### MicroRNA transfection

Transfections with Pre-miR^™^ microRNA precursor pre-miR-223-5p (Prod. ID: PM12672) were performed with Lipofectamine 2000 (Invitrogen, USA) at 50 nM final concentration.

### qRT-PCR quantification of miR-223-5p on SW-962 cells

Total RNA was purified from SW962 cell line before and after transfections using TRIZOL Reagent (Invitrogen) according to manufacturer's instructions. cDNA synthesis was performed using TaqMan MicroRNA Reverse Transcription Kit and miR-223-5p quantification was done using TaqMan microRNA Assays Kit (AssayID:000512). qRT-PCR experiment was conducted in triplicates, normalized to U6 (Cat. #4427975) and carried out on the 7900 ABI system (Applied Biosystems). Results are presented based on 2^-ΔΔCt^ method [71].

### Cell proliferation assay

Briefly, 5 × 10^4^ SW962 cells per well were seeded into 24-well plates, incubated for 24 h, and transfected with mimetic oligonucleotides. Cells were harvested at four time points after transfection (24, 48, 72 and 96 hours) and measured automatically using Vi-cell XR Cell viability analyzer (Beckman Coulter, Miami, FL, USA), generating cell proliferation and cell viability curves. The experiments were performed in triplicate.

### Wound-healing assay

Two hundred thousand SW962 cells were plated in 6-well plates 24 hours after transfection with mimetics. To discard a proliferation component of cell migration, serum-free medium containing 10 ug/ml mitomycin C (Sigma-Aldrich, Cat#M0503) was added one hour prior to scraping the monolayer. Cells were washed with PBS to remove the ones released in the process and 2mL of RPMI medium without serum were added to each well. After total confluence of the cells, a vertical scar was done with a P200 pipette applying an angle of 30°. Cells were incubated and photos were taken at 24, 48, 72 and 96 hours after the scar on 20× magnification at the Nikon confocal microscope LiveScan FSC (Nikon Inc. Instruments). The area corresponding to each scar was obtained with Image J software (Wayne Rasband, National Institutes of Health, Bethesda, MD), after manual selection. The assay was performed in triplicates and three cell passages were used in order to allow biological replicates.

### Invasion assay

For invasion assay, 2 × 10^5^ cells were plated in the top chamber of a Matrigel-coated membrane (Corning, USA). After 24 hours, cells were put on a serum-free condition for 6 hours and medium containing serum was added to the lower chamber, as a chemo attractant. After 24 hours, cells were washed with PBS, fixed with 4% paraformaldehyde, followed by DAPI staining (1:500). Cells from the upper chamber were removed using a cotton swab and invading cells were photographed using inverted microscope in 8 different fields. Quantification was performed automatically using Image J software.

### Identification of targets for hsa-miR-223-5p

miRWalk, Diana mT and miRanda [72–74] gene prediction algorithms were searched attempting to find 3′UTR mRNA regions containing conserved binding sites for hsa-miR-223-5p. The chosen target was further evaluated – both mRNA and protein – in order to check its putative role upon suppression by hsa-miR-223-5p in vulvar cancer cell line SW962.

### qRT-PCR quantification for *TP63*

RNA was extracted from SW962 cell line using TRIZOL Reagent (Invitrogen) as previously mentioned. cDNA synthesis was performed from 1,000 ng RNA using SuperScript^®^ VILO cDNA Synthesis Kit (Life Technologies). *TP63* was quantified using TaqMan^®^ Gene Expression Assay for TP63 (Hs00186613_m1) and TaqMan^®^ Fast Advanced Master Mix (Life Technologies). Experiments were conducted in triplicates, normalized to Human *HPRT1* Endogenous Control (Cat. #4326321E) and carried out on the 7500 Fast Real Time PCR system (Applied Biosystems).

### Western blot

Protein expression of p63 and α-Tubulin were assessed in SW962 vulvar cancer cell line after 24 hours of transfection with oligonucleotides through Western blot analysis. Cell lysates from transfected and non-transfected cells were obtained using RIPA lysis buffer supplemented with protease and phosphatase inhibitors cocktail (Sigma-Aldrich, MO, USA). Total protein quantification was performed through Bradford standard curve method. 20 ug protein extract was separated by a 10% SDS-PAGE and transferred onto a polyvinylidene difluoride (PVDF) membrane (GE Healthcare, USA). After blocking with 5% non-fat milk, membranes were incubated with antibodies anti-p63 (1:200 dilution, Novocastra NCL-p63) or anti-α-Tubulin (1:1000 dilution, Sigma-Aldrich DM1A) overnight at 4°C. Membranes were then incubated for one hour with Anti-mouse IgG, HRP-linked secondary antibody at room temperature (1:3000 dilution, Cell Signaling#7076S). Immunoreactivity was detected using Amersham ECL Prime Western blotting detection reagent (GE, Healthcare, USA) according to the manufacturer's instructions. Bands were scanned using UVITEC Alliance LD (Uvitec, Cambridge, UK) equipment and quantified using Image J software and p63 densitometric values of protein expression were normalized to the corresponding α-Tubulin protein levels.

### Immunofluorescence

SW962 cells (100,000 cells) were plated on glass coverslips, fixed with 4% paraformaldehyde and permeabilized with 0.25% Triton ×-100 (Sigma-Aldrich). Cells were incubated with primary antibody against p63 (1:100 dilution, Novocastra NCL-p63), Donkey anti-Mouse IgG (H + L) Secondary Antibody, Alexa Fluor^®^ 488 conjugate (Life Technologies) and DRAQ5 far-red fluorescent dye (1:500 dilution, Cell Signaling Technology, MA, USA). Coverslips were mounted with FluorSave mounting reagent (Calbiochem, UK) and then analyzed under Leica TCS SP5 II Confocal Microscope (Leica Microsystems).

### Samples from patients

Eighteen patients diagnosed with vulvar squamous cell carcinomas were randomly selected from the Department of Pathology, AC Camargo Cancer Center, from 1980 to 2009. Formalin-fixed paraffin-embedded (FFPE) tissues of these patients as well as their medical records were collected. Cases with no adequate available FFPE tissue or missing clinical data were excluded as well as those patients who went through any neoadjuvant therapy. Also, cases from which RNA extraction yielded poor quality material were excluded. HPV infection was tested as previously mentioned [75]. Seven normal tumor-adjacent vulvar skin samples were also collected and used as non-tumor control for qRT-PCR normalization purposes. Approval for this study was obtained from AC Camargo Ethics Committee Review Board (Approval number 1622/11) and all experiments were conducted in accordance with the Helsinki Declaration and other relevant guidelines. Important clinicopathological data from the patients are demonstrated on Supplementary Table S2.

### Immunohistochemistry (IHC)

Immunohistochemistry was performed automatically on Benchmark platform (Ventana Medical Systems, USA) for p63 (Ventana 4A4 Clone, ready-to-use antibody) in whole slide tissues. Appropriate positive control was used (tonsil) and negative control was obtained as omitting the primary antibody.

Slides were digitalized on APERIO^®^ digital scanner (Leica Biosystems) and five random areas for each case were selected for automated analysis through a nuclear algorithm. Immunohistochemical expression of p63 was quantitatively obtained based on both the percentage of stained cells (varying from 0 to 100% of cells) and staining intensity (0, 1, 2 or 3), generating a Histologic Score (HScore). Immunoreactivity for p63 was recorded as negative if HScore < 150 and positive when ≥ 150, which has been previously shown to demonstrate more accurate results [76, 77].

### qRT-PCR quantification of miR-223-5p on FFPE tissues

Total RNA was purified from FFPE tissues from 18 patients and 7 normal samples (constituting a normal pool) using RecoverAll Total Nucleic Acid Isolation Kit (Ambion) according to manufacturer's instructions. cDNA synthesis was performed using TaqMan MicroRNA Reverse Transcription Kit and miR-223-5p quantification was done using TaqMan microRNA Assays Kit (AssayID:000512). qRT-PCR experiment was conducted in triplicates, normalized to Human HPRT Endogenous Control (Cat. #4326312E) and carried out on the 7900 ABI system (Applied Biosystems).

### Statistical analysis

Statistical analysis was held on GraphPad Prism version 5.01 for Windows, (GraphPad Software, San Diego California USA). Experiments were performed three times, so the results are presented as mean (± SD). ANOVA with Bonferroni corrections, Fisher's exact test, Student *t* test, Mann-Whitney and Kaplan-Meier tests were performed when appropriate. Results were considered as statistically significant when *p* < 0.05.

## SUPPLEMENTARY MATERIALS TABLES


