# Supplementary material for: MiR-223-5p works as an oncomiR in vulvar carcinoma by TP63 suppression
Source: Oncotarget. 2016 Jun 23;7(31):49217–31. doi: 10.18632/oncotarget.10247 (PMC5226502; doi:10.18632/oncotarget.10247)
Supplement: Supplementary file 1 [file oncotarget-07-49217-s001.pdf]

MiR-223-5p works as an oncomiR in vulvar carcinoma by *TP63* suppression

Supplementary Materials

Supplementary Table S1: Immunohistochemistry for p63 versus main clinicopathological features from the patients with vulvar carcinoma

Immunohistochemistry for p63 versus clinicopathological features					
	p63 negative		p63 positive		*p* value
	n	%	*n*	%	
**Immunohistochemistry**	11	*61,11*	7	*38,89*	-
**Tumor Grade**					
SCC *Grade I*	3	*60*	2	*40,00*	*p* = 0.99
SCC *Grade II*	5	*62,50*	3	*37,50*	
SCC *Grade III*	3	*60*	2	*40,00*	
**HPV infection**					
Negative	7	*63,64*	4	*36,36*	*p* = 1.000
Positive	4	*57,14*	3	*42,86*	
**Depth of tumor invasion**					
Superficial/Medial dermis	2	*28,57*	5	*71,43*	***p* = 0.0491 (*)**
Deep dermis/Subcutaneous	9	*81,82*	2	*18,18*	
**Nodal metastasis***					
Absent	4	*80*	1	*20*	*p* = 1.000
Present[a]	5	*100*	0	*0*	
**Perineural invasion***					
Absent	6	*46,15*	7	*53,85*	*p* = 0.2125
Present	3	*100*	0	*0*	
**Vascular invasion***					
Absent	7	*53,85*	6	*46,15*	*p* = 0.6029
Present	3	*75*	1	*25*	

*Some data were lost.
a Presence of at least one metastatic lymph node.

Supplementary Table S2: Main clinicopathological features from the patients with vulvar carcinoma

Clinicopathological features		
**Age**		
Median	73 years (SD = 17.36)	
Range	15–89	
	n	%
**Tumor Grade**		
SCC Grade I	5	27.78
SCC Grade II	8	44.44
SCC Grade III	5	27.78
**HPV infection**		
Negative	11	61.11
Positive[a]	7	38.89
HPV18	3	42.86
HPV33	3	42.86
HPV16	2	28.57
HPV67	1	14.28
**Depth of tumor invasion**		
Superficial/Medial dermis	7	38.89
Deep dermis/Subcutaneous	11	61.11
**Nodal metastasis***		
Present[b]	5	27.77
Absent	5	27.77
**Perineural invasion***		
Present	3	16.66
Absent	13	72.22
**Vascular invasion***		
Present	4	22.22
Absent	13	72.22

*Some data were lost.
[a]HPV co-infection was observed in one case.
[b]Presence of at least one metastatic lymph node.
